# Adiposity-dependent adipokine-immunoglobulin interplay in mature breast milk

**DOI:** 10.3389/fendo.2026.1873967

**Published:** 2026-08-28

**Authors:** Jolanta Lis-Kuberka, Magdalena Humaj-Grysztar, Anna Apanasewicz, Maja Matyas, Anna Ziomkiewicz, Magdalena Orczyk-Pawiłowicz

**Affiliations:** 1 Division of Chemistry and Immunochemistry, Department of Biochemistry and Immunochemistry, Wroclaw Medical University, Wroclaw, Poland; 2 Institute of Nursing and Midwifery, Faculty of Health Sciences, Jagiellonian University Medical College, Krakow, Poland; 3 Department of Anthropology, Hirszfeld Institute of Immunology and Experimental Therapy, Polish Academy of Sciences, Wroclaw, Poland; 4 Jagiellonian University, Doctoral School of Exact and Natural Sciences, Krakow, Poland; 5 Laboratory of Anthropology, Institute of Zoology and Biomedical Research, Jagiellonian University, Krakow, Poland

**Keywords:** adipokines, human milk, immunoglobulins, lactoferrin, maternal body fat, mature milk

## Abstract

**Introduction:**

Excessive maternal body fat (BF) accumulation promotes low-grade inflammation, altering milk adipokine and immunoglobulin profiles and potentially contributing to intergenerational immunometabolic programming of offspring. This observational study focused on mothers with higher BF and evaluated associations between adipokines and immune components in mature milk.

**Methods:**

At 5 months of lactation (N = 59), concentrations of lactoferrin (LF), immunoglobulins (S-IgA, IgG, IgM), CRP, adipokines (leptin, adiponectin, and ghrelin), and the leptin-to-adiponectin ratio (LAR) were quantified in milk samples using an immunoenzymatic assay. ANCOVA models assessed associations between maternal BF categories (high vs normal) and milk adipokines and immunological components.

**Results:**

Mature milk from mothers with higher BF showed significantly higher concentrations of CRP (8.00 vs 3.44 μg/L), leptin (0.41 vs 0.14 μg/L), LAR (0.13 vs 0.05), and IgM (2.78 vs 1.59 mg/L) than the normal BF group. ANCOVA models revealed positive associations between milk leptin and LF (β=0.51, *p* = 0.022) and S-IgA (β=0.47, *p* = 0.014), as well as LAR and LF (β=0.37, *p* = 0.032). No significant differences in adiponectin, ghrelin, LF, S-IgA, or IgG concentrations were observed between the analyzed groups. Elevated maternal BF is associated with higher levels of breast milk inflammatory markers (CRP, leptin), LAR, and IgM, suggesting metabolically induced inflammation.

**Discussion:**

Leptin-immunoactive component associations (S-IgA, LF) offer novel insights into milk bioactivity. These findings highlight the role of metabolic-immunological milk components in shaping health trajectories for infants of mothers with elevated BF.

## Graphical Abstract

**Figure:**
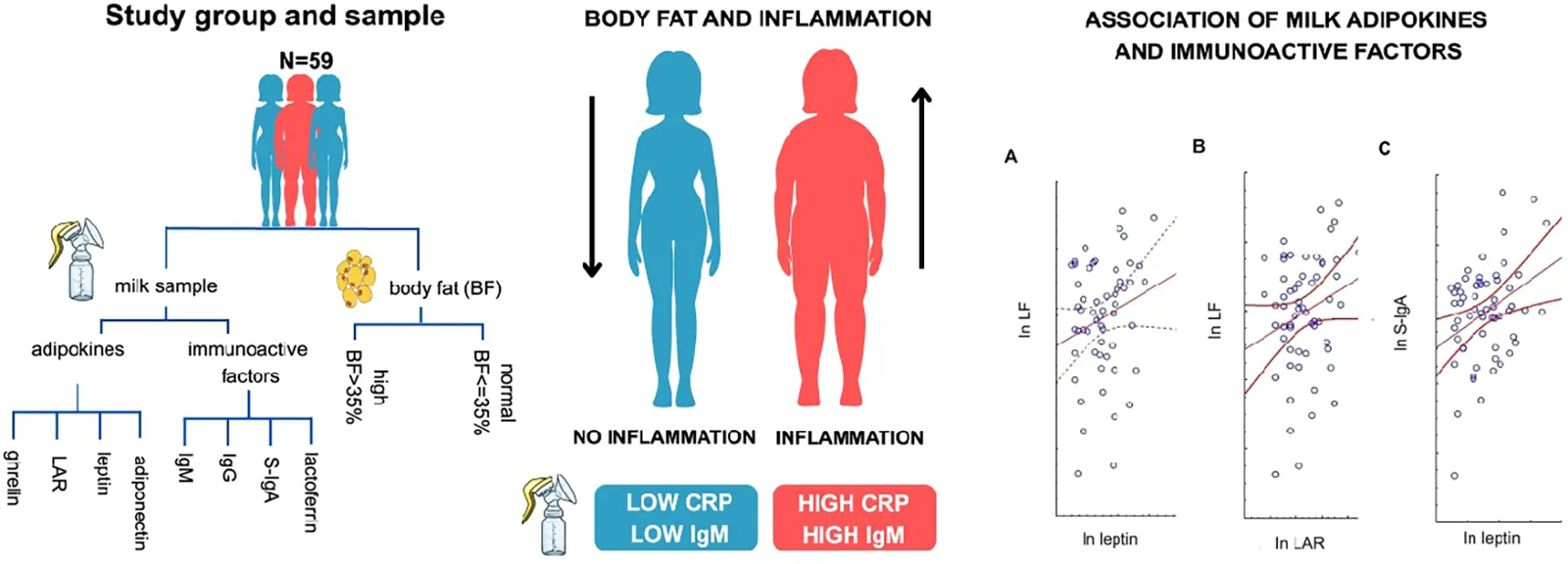

## Highlights

- Elevated maternal body fat (BF) is associated with higher concentrations of mature-milk CRP, leptin, and the leptin-to-adiponectin ratio (LAR), indicating low-grade inflammation linked to abnormal fat accumulation.
- Mature milk leptin is positively associated with the immunological component S-IgA, suggesting coordinated immunometabolic regulation through the gut-mammary axis.
- The association between leptin and lactoferrin (LF) in maternal milk suggests a coordinated immunometabolic adaptation to subclinical inflammation among mothers with higher body fat.
- Mature milk IgM levels are significantly higher in mothers with elevated BF, potentially due to leptin-mediated effects on B-cell class-switch recombination and to natural IgM derived from adipose tissue.

## 1 Introduction

Human milk is considered the gold standard for infant nutrition. It provides adequate energy and essential nutrients, along with a wide range of bioactive components that support growth, immunity, and metabolic programming. The composition of breast milk is highly dynamic and varies with maternal and infant factors. Recent studies have highlighted lactation stage, diet, age, physical and psychological health, and nutritional status as maternal characteristics that can alter milk composition (1–7). These factors can affect the levels of macronutrients, vitamins, and immune components (8–10). Breast milk is rich in immunoglobulins and bioactive peptides, and maternal diet influences fatty acid and vitamin content. Some infant characteristics, such as gestational age at birth, health status, sex, and feeding behaviours, also affect milk composition (1, 11, 12).

Human milk undergoes a well-described maturation process, from colostrum through transitional milk to mature milk (13). Colostrum is produced during the first days postpartum (from birth to 5–6 days) as a yellowish fluid rich in proteins (14–16 g/L), which includes immunoglobulins (mainly secretory IgA) (5.4 g/L) and lactoferrin (5g/L). Transitional milk is produced from days 7 to 14/15, followed by mature milk from approximately 2 weeks onward (4). The concentration of fat and lactose is relatively low compared with subsequent stages of lactation (26 g/L and 56 g/L, respectively) (14–20). As lactation progresses to the transitional and then mature milk stages, total protein (7–10 g/L) and S-IgA (1.3 g/L) concentrations decrease, while lactose and fat concentrations increase (37 g/L and 69 g/L, respectively), reflecting a shift from predominantly immunoprotective to more energy- and growth- supporting offspring nutrition (14–20).

Among a wide array of bioactive milk components, immunoglobulins and adipokines play crucial roles in shaping the infant’s immune and metabolic programming. Secretory IgA (S-IgA) is the predominant immunoglobulin and serves as the first line of defence at mucosal surfaces by providing antigen-specific immune exclusion. It supports homeostasis and protects the breastfed infant during a period of developmental immaturity of the immune system (4, 21, 22). IgG and IgM, which occur at lower concentrations, contribute to primary immune defence by neutralizing pathogens before the infant’s endogenous antibody synthesis reaches an optimal level (23). The concentrations of milk immunoglobulins depend on several factors, including lactation stage, gestational age, maternal health, and environmental influences, reflecting a highly adaptive immune composition (4, 22, 24, 25).

Human milk also contains lactoferrin (LF), which sequesters free iron, inhibiting bacterial, viral, and fungal growth while promoting beneficial gut microbiota, including *Bifidobacterium* and *Lactobacillus* (26, 27). It modulates immune responses by enhancing epithelial barrier function, reducing inflammation through cytokine regulation, and supporting neutrophil activity (28–30). Mean LF values range from 4.90 to 5.02 g/L, with the highest concentration between 12 and 24 months postpartum (22). Moreover, Wu et al. (31) reported that milk from mothers suffering from obesity contains a higher LF concentration than milk from women of normal weight.

In addition to adaptive and innate immunological components, human milk contains adipokines, which are primarily secreted by adipose tissue and involved in appetite regulation and in lipid and glucose metabolism (32–34). The concentration of adipokines in milk may be modulated by maternal BMI, body fat (BF), and metabolic status (6, 7, 35, 36). The stage of lactation also influences adipokine levels, although research evidence rarely considers mature milk beyond 3 months postpartum (37, 38), thereby affecting the maturation of the infant metabolic-endocrine axis (33, 39, 40).

The most frequently studied adipokines are leptin and adiponectin. The primary distinction between these hormones lies in their association with adiposity. Leptin levels generally increase with higher BF, whereas adiponectin concentrations are typically lower in women with higher BF (41–43). Moreover, adiposity distribution influences circulating adiponectin levels. Specifically, individuals suffering from obesity and a higher subcutaneous adipose tissue (SAT) to visceral adipose tissue (VAT) ratio may have higher adiponectin concentrations. The waist-to-hip ratio (WHR) is a commonly used marker of adiposity distribution (44, 45).

Pregnancy-related physiological changes in maternal body composition include a 30 to 50% increase in BF accumulation. Maternal metabolic adaptation to these changes includes a progressive rise in leptin concentrations, further driven by placental production. Adiponectin levels remain relatively stable or slightly elevated early on, before declining significantly in later trimesters (46–50). These shifts enable maternal energy storage during early pregnancy and support fetal growth later on. Excessive gestational weight gain in mothers can alter the pattern of adipokine secretion, promote chronic low-grade inflammation, and contribute to the pathogenesis of metabolic disorders (41, 50–54). Maternal diet has also been identified as an important factor related to adipokine levels, with certain dietary patterns leading to increased (55–58) or decreased adiponectin concentrations (59–62). These dietary patterns may, in turn, modulate adipokine profiles in breastmilk and potentially affect the child’s postnatal growth trajectories (63–65). Milk from mothers with high BF exhibits higher leptin and lower adiponectin concentrations, a pattern linked to early-life metabolic programming of excessive weight gain and obesity in offspring (36, 66–69). Cortés-Macías et al. (70) demonstrated that variations in milk adipokine and cytokine profiles correspond with different infant growth trajectories during the first year of life.

Animal models demonstrate that adipokines regulate lactogenic hormone action, thereby influencing immune responses within the mammary gland (71–73). Adiponectin inhibits inflammation by blocking NF-κB activation and reducing pro-inflammatory cytokines such as TNFα, IL-6, and IL-18 (74, 75). In a rodent model, leptin and adiponectin supplementation during the suckling period alters intraepithelial lymphocyte subsets and reshapes the gut microbiota, highlighting their role in early intestinal immune maturation (76). The higher pro-inflammatory milieu affects C-reactive protein (CRP) concentration in maternal circulation. Moreover, CRP levels in breast milk are higher in mothers with overweight/obesity than in normal-weight women, and these levels decrease as lactation progresses (77, 78). Similarly, inflammation of the mammary gland, as described in the mastitis spectrum, alters CRP concentrations in both maternal serum and milk. Notably, increased CRP levels can be found not only in the milk of the inflamed breast, but also in the milk from the asymptomatic breast of the same lactating woman (79, 80).

Investigating interactions between maternal metabolic status and milk immunoactive factors is essential for identifying biomarkers of neonatal metabolic programming and for developing interventions to optimize maternal lactation and promote healthy infant development. The present study examines how maternal BF (amount and distribution) is associated with key milk immunometabolic factors, including markers of inflammation (CRP), adipokines (leptin, adiponectin, and ghrelin), immunoglobulins (S-IgA, IgG, and IgM), and lactoferrin. This examination will offer novel insights into the immunometabolic composition of milk (81, 82).

We hypothesized that high maternal body fat would be associated with a more pro-inflammatory and metabolically unfavourable profile of mature human milk. Specifically, we expected higher CRP and leptin concentrations and lower adiponectin concentrations in milk from mothers with high BF compared with those with normal BF. We also expected these adipokine differences to be accompanied by alterations in milk immune components, including immunoglobulins and lactoferrin.

## 2 Materials and methods

### 2.1 Participant selection criteria

The study sample consisted of mothers from Wroclaw, south-western Poland, who participated in the Mum’s Milk Plus project (2). Exclusively breastfeeding mothers volunteered in response to advertisements on the radio, in newspapers, on social media (Facebook groups for pregnant and lactating women), and at local gynecological clinics and antenatal birth schools. The inclusion criteria for mothers were: a) at least 18 years old; b) not taking any steroid treatment during lactation; c) not smoking or drinking alcohol; d) free from any self-reported chronic diseases; e) having a single, uncomplicated pregnancy; f) exclusively breastfeeding infants from birth until at least the time of study recruitment. The inclusion criteria for infants were: a) born at term (gestational age ≥37 weeks) with appropriate birth weight for gestational age (not less than 2500g); b) free from congenital diseases. Neither maternal nor infant food allergies were included as specific exclusion criteria. A total of 160 eligible pairs (mother and infant) were enrolled in the Mum’s Milk Plus project. For the present analysis, data from women with high body fat (BF>35%, N = 31) and randomly sampled control women (BF ≤ 35%, N = 28) were included to examine the associations of interest. Thus, the final analytical sample consisted of 59 women.

### 2.2 Study procedure

Women were asked to participate in three study meetings. The first and second meetings occurred when the children were 5 months old, and the third when the children were 12 months old. In this study, we analyzed data from the first two meetings, focusing on mature milk, which is comparatively less investigated and more feasible to collect than colostrum and early transitional milk. The 5-month postpartum time point was selected to assess breast milk composition during established lactation and before the introduction of complementary feeding (83, 84).

During this period, infants experience rapid growth, ongoing immune and gut maturation, and substantial metabolic development. Therefore, variation in milk inflammatory, metabolic, and immune components, including CRP, adipokines, immunoglobulins, and lactoferrin, may be particularly relevant to the infant’s early nutritional and immunological environment.

During the first meeting, we collected anthropometric measurements from mothers and information on pregnancy and birth outcomes. During the second meeting, we collected anthropometric measurements of infants and data on maternal and infant socioeconomic status, self-reported health, and maternal daily food intake. We also collected mature milk samples to analyze adipokines, CRP, lactoferrin, and immunoglobulin levels. All mothers received information about the study’s purpose and course and provided informed written consent to participate on their own behalf and on behalf of their children. In accordance with the Declaration of Helsinki, mothers could withdraw from the study at any time without legal or financial consequences. The study protocol was approved by the Bioethical Committee of the Lower Silesian Medical Chamber in Wroclaw (approval identification number 1/NT/2016, obtained on 10.02.2016).

#### 2.2.1 Daily food intake

Mothers were asked to report all food items (dishes, drinks, and supplements) consumed on three selected weekdays (including one weekend day) using daily logs. They assessed the size of each food item or dish using sample albums provided with the logs. When a food item was not in the album, mothers were asked to copy the product’s weight from the packaging or weigh the item on a kitchen scale and record it in the logs. A trained study assistant entered the type and size of each dietary item into the Diet 5D computer program, developed by the Polish National Health Institute, to calculate the content of selected macro- and microelements. The software database is based on Polish food composition tables and national dietary reference standards (85) and has been widely used in nutritional research and clinical practice in Poland (86, 87). Maternal intake of fat and saturated fatty acids (maternal SFA intake) was expressed in grams per day. Data from the three days were averaged.

#### 2.2.2 Anthropometric measurements

Maternal anthropometric measurements were taken at the first study meeting, whereas infant measurements were obtained at the subsequent meeting, one week later. All measurements were repeated three times and averaged. Maternal measures included body weight and fat percentage, measured with the Tanita SC-240 MA scale (0.1 kg for weight and 0.1% for BF), and height measured with a stadiometer (0.1 cm accuracy). These data were used to calculate BMI using the standard formula. Breast, under breast, waist, and hip circumferences were measured with an anthropometric tape (0.1 cm accuracy). Based on these results, the breast index (breast circumference/under breast circumference) and waist-to-hip ratio (WHR=waist circumference/hip circumference) were calculated. WHR was treated as an indicator of BF distribution (88, 89).

In infants, body weight was measured with an analog hospital scale (0.1 kg accuracy), length with the Seca measuring board model 417 (0.1 cm accuracy), and head circumference with an anthropometric measuring tape (0.1 cm accuracy). In addition, birth characteristics, including gestational age, birth weight, length, and head circumference, were obtained from the child’s health record.

### 2.3 Mature milk sample collection and analysis

Mothers collected a single milk sample in sterile containers using the Medela Symphony breast pump (Medela AG, Switzerland) under the supervision of the trained research assistant in the study room. Samples were collected between 1 and 1.5 hours after the second feeding of the day to standardize collection time relative to possible diurnal changes in milk composition (90, 91). Mothers were instructed to pump from one breast until it was empty (92) to avoid variability associated with the breastfeeding phase. Because research showed no significant difference in mature milk composition between the left and right breasts, mothers were free to choose which breast to collect milk from (93). Immediately after collection, milk samples were aliquoted into multiple containers of varying volumes (1.5, 10, and 15 mL) according to the planned analyses. At 5 months postpartum, the minimum amount of milk collected from a mother was 60 mL, and aliquots not used in the present study were stored at -80 °C for other analyses conducted within the Mum’s Milk project.

The soluble fraction of breast milk (skim milk aqueous phase) was obtained by centrifugation at 3500 × g for 35 min at 4 °C, followed by removal of the fat layer and cellular debris. Aliquots of skim milk were then stored at -20 °C for subsequent assays (2, 94, 95).

### 2.4 Determination of lactoferrin concentration

The concentration of LF in skim mature milk samples was determined using an enzyme-linked immunosorbent assay (ELISA) following previously described procedures with minor modifications (2, 22, 95). All analyses were performed in microtiter plates (Nunc International, Naperville, IL, USA). Standard curves were constructed using purified human milk lactoferrin (Sigma Aldrich, St. Louis, MO, USA) within a detection range of 1.56 to 50 ng per 100 µL.

Mature milk samples were analyzed at three dilutions (10,000-, 25,000-, and 50,000-fold). For the assay, rabbit anti-human lactoferrin phosphatase-labeled antibodies (Jackson ImmunoResearch Europe Ltd., Ely, UK) were used. The enzymatic color reaction was developed using a phosphatase substrate, and absorbance was measured at 405 nm with a 630 nm reference filter on a Synergy LX Multi-Mode Reader (BioTek Instruments, Inc., Winooski, VT, USA). Each measurement was performed in duplicate.

The intra- and inter-assay coefficients of variation for the LF-ELISA were 4.3% and 3.6%, respectively.

### 2.5 Determination of S-IgA, IgG, and IgM concentrations

The concentrations of secretory IgA (S-IgA), IgG, and IgM in the soluble fraction of mature human milk samples were measured by ELISA, as previously described (2, 22, 95).

The following antibodies were used in the tests: F(ab’)_2_ fragments of goat anti-human IgG for IgG, rabbit anti-human IgM for IgM (both from Jackson ImmunoResearch, Europe Ltd., Ely, UK), and mouse monoclonal anti-secretory component IgA for S-IgA (Sigma, St. Louis, MO, USA). Standard curves were generated using human serum IgG and human IgM (both from Jackson ImmunoResearch, Europe Ltd., Ely, UK) and human colostrum IgA (Sigma, St. Louis, MO, USA), respectively. For detection, alkaline phosphatase (AP)- and horseradish peroxidase (HRP)-labeled secondary antibodies were used: rabbit anti-human IgG Fcγ fragment-specific AP-conjugated antibodies for IgG, goat anti-human IgM HRP-conjugated antibodies for IgM (both from Jackson ImmunoResearch, Europe Ltd., Ely, UK), and goat anti-mouse IgG HRP-conjugated antibodies for S-IgA (Sigma, St. Louis, MO, USA).

The milk sample was analyzed at three dilutions optimized for each immunoglobulin class: 25,000-, 50,000-, and 100,000-fold for S-IgA; 500-, 1,000-, and 1,500-fold for IgG; and 50-, 100-, and 250-fold for IgM. All measurements were run in duplicate. Enzymatic reactions for AP and HRP were developed with appropriate substrates, and absorbance was measured at 405 nm (reference 630 nm) for AP and at 492 nm (reference 630 nm) for HRP using a Synergy LX Multi-Mode Reader (BioTek Instruments, Inc., Vermont, USA). Immunoglobulin concentrations were calculated from the standard curves and expressed in appropriate units. The intra- and inter-assay coefficients of variation were as follows: for S-IgA, 2.8% and 3.3%, for IgG, 3.2% and 4.8%, and for IgM, 4.1% and 3.6%, respectively.

### 2.6 Determination of C-reactive protein concentration

The concentration of C-reactive protein (CRP) in the soluble fraction of milk samples was measured by ELISA following a modified protocol (5, 96). Microtiter plates (Nunc International, Naperville, IL, USA) were coated with anti-CRP antibody (IgG fraction; Sigma-Aldrich, St. Louis, MO, USA) at a 1:5000 dilution in Tris-buffered saline (TBS; pH 8.0) and incubated for 2 h at 37 °C. Wells were subsequently blocked with TBS (pH 8.0) containing 0.1% Tween-20 and 0.2 mg% bovine serum albumin (BSA) for 1 h at 37 °C, followed by overnight incubation at 4 °C. For analysis, 100 μL of 1:10 diluted mature milk sample or CRP standard solutions (0.04-2.5 ng/100 μL; Sigma-Aldrich) in Tris-HCl buffer (pH 8.0) with 0.05% Tween-20 were added to the wells and incubated for 1 h at 37 °C. Detection was performed using a horseradish peroxidase (HRP)-conjugated goat anti-CRP antibody (Abcam, Cambridge Biomedical Campus, UK) at a 1:50,000 dilution in TBS with 0.05% Tween-20. The enzymatic reaction was developed for 10 min at room temperature in the dark using o-phenylenediamine (Calbiochem, Denmark) in 0.1 M citrate buffer (pH 5.0) containing H_2_O_2_. The reaction was stopped with 12.5% H_2_SO_4_, and absorbance was measured at 492 nm (reference 630 nm) using a Synergy LX Multi-Mode Reader (BioTek Instruments, Vermont, USA). All samples were analyzed in duplicate. The intra-assay and inter-assay coefficients of variation were 1.0% and 5.2%, respectively.

### 2.7 Determination of leptin, adiponectin, and ghrelin concentration

The concentrations of adiponectin and leptin in human milk were measured using commercial ELISA kits (Biorbyt, Cambridge, United Kingdom) after prior optimization of the assays for mature milk sample analysis (6). The assay detection ranges were 31.25–2000 pg/mL for adiponectin and 62.50–4000 pg/mL for leptin. All skim milk samples were analyzed in duplicate. Samples were diluted 1:10 for adiponectin and 1:2 for leptin. The intra-assay and inter-assay coefficients of variation were 1.7% and 5.1% for adiponectin and 1.1% and 3.2% for leptin, respectively.

Ghrelin concentration in mature milk was measured using a commercial ELISA kit (EIAab Science Co., Ltd., Wuhan, China) after prior adaptation for milk samples. Skim milk samples were directly transferred to the assay plates. The assay detection range was 0.156–10 ng/mL. All samples were run in duplicate. The intra-assay and inter-assay coefficients of variation were 4.3% and 9.1%, respectively.The leptin-to-adiponectin ratio (LAR), a composite biomarker commonly used to assess adipose tissue dysfunction and metabolic risk, was calculated as the ratio of leptin-to-adiponectin concentration (LAR=leptin [μg/L] / adiponectin [μg/L]) (6, 97, 98).

### 2.8 Statistical analysis

Normality of the distributions of the dependent variables (immunoglobulins, lactoferrin, adipokines, and CRP) and homogeneity of variance were assessed using the Shapiro-Wilk test (normality) and Levene’s test (homogeneity). Concentrations of immunoglobulins and lactoferrin were log-transformed (ln) before further analysis to improve homogeneity of variance.

Women were grouped by body fat (BF) level, with normal and high BF defined by a 35% cutoff (99). Although BMI or gestational weight gain are more commonly used indicators during pregnancy, maternal BF provides a more direct estimate of maternal adiposity and body composition than BMI, which does not distinguish between fat and lean mass. Given that adipokines are secreted primarily by adipose tissue and may influence immunoglobulin production and secretion, we considered BF a more physiologically relevant measure for examining the adipokine-immunoglobulin interplay in human milk.

WHR values were narrowly distributed (mean WHR 0.75 overall; 0.73 in women with normal body fat and 0.77 in those with higher body fat), and only a minority of participants reached the conventional cut-off for abdominal obesity (WHR≥0.8). The WHR cut-off was based on the study sample median (Me=0.75). To obtain balanced subgroups and a data-driven threshold, we used the sample median WHR (0.75) to classify participants into lower- and higher-WHR categories. Women with WHR values equal to or lower than the median were included in the normal WHR group, whereas those with WHR values above the median were included in the higher WHR group.

Differences in maternal and infant characteristics, as well as in mature milk adipokines and immunoactive factors, between mothers with normal and high BF were assessed using Student’s t-test when variable distributions were normal, or the Mann-Whitney U test when distributions deviated from normality.

The association between maternal body fat, mature milk adipokines, and immunoactive factors was tested using General Linear Models (GLMs). Specifically, one-way ANCOVA models assessed the effects of maternal BF (categorical predictor) and adipokine levels (continuous predictor) on milk immunoactive factor levels, while controlling for infant gestational age and maternal SFA intake. Separate models were run for each immunological mature milk component (S-IgA, IgM, IgG, and lactoferrin) as the dependent variable. Each model included BF as a binomial independent variable (normal vs. high). WHR models were built similarly. WHR was treated as a binomial independent variable (normal vs. high), whereas other dependent and independent variables remained unchanged.

Statistical analyses were performed using StatSoft Statistica 13.3 software. The statistical significance level was established at *p* < 0.05.

## 3 Results

### 3.1 Study group characteristics

The study group included 47.46% women with normal BF and 52.54% with high BF. The mean age of the study participants was 31.37 years (SD = 4.758). They had a relatively good socioeconomic situation, with financial satisfaction rated at 5 out of 7 points (IQR = 1). Also, 88.14% of mothers had at least a college degree. 61.02% of women were primiparous, and 47.46% delivered a baby vaginally. Among infants, 67.80% were boys. The mean age of infants was 4.7 months (SD = 0.550), with a range from 4.5 to 5.8 months. Their average birth weight was 3576.1 g (SD = 426.279) and length 55.3 cm (SD = 2.930).

As expected, women with high BF had higher BMI (26.48 vs 21.15; *p* < 0.001) and BF mass (35.50 kg vs 14.50 kg; *p* < 0.001). Although the breast index did not differ between groups (*p* = 0.519), WHR was significantly higher in the high-BF group than in the normal-BF group (0.77 vs 0.73; *p* = 0.001). Moreover, women with high BF had higher fat intake (80.51 vs 63.82; *p* = 0.015), primarily saturated fatty acids (SFA; 32.12 vs 22.22; *p* = 0.001), than women with normal BF. In addition, infants of women with high BF had higher birth weight (3687.1 g vs 3453.2 g; *p* = 0.034) but did not differ in birth length compared with infants of women with normal BF. Other maternal and infant characteristics did not differ between the high- and normal-BF groups (**Table 1**).

**Table 1:** Maternal and infant characteristics in the total analytic sample and by maternal body fat category.

Maternal and Infants Characteristics	All N=59	Normal BF N=28	High BF N=31	*p*
Mothers				
Age [years] ^1^	31.37 (4.76)	31.85 (4.64)	30.95 (4.90)	0.476
Financial satisfaction^1^	5.00 (1.00)	5.00 (1.00)	5.00 (1.00)	0.266
Education [% highly educated]^2^	88.14	89.29	87.10	0.941
Parity [% primiparous]^2^	61.02	60.71	61.29	0.964
Mode of delivery [% vaginal delivery]^2^	47.46	39.29	54.84	0.181
BMI [kg/m^2^]^1^	25.15 (5.66)	21.15 (2.79)	26.48 (3.20)	<0.011*
Fat mass [kg]^1^	21.00 (11.60)	14.50 (6.650)	25.50 (10.90)	<0.001*
Breast index^1^	1.20 (0.07)	1.21 (0.082)	1.19 (0.03)	0.519
WHR^1^	0.75 (0.07)	0.73 (0.032)	0.77 (0.12)	0.001*
Vegetarian [% yes] ^2^	1.69	0.00	3.22	0.290
Maternal fat intake [g]^1^	69.34 (49.19)	63.82 (24.57)	80.51 (57.75)	0.015*
Maternal SFA intake[g]^1^	27.66 (22.20)	22.22 (11.90)	32.12 (27.55)	0.001*
Infants				
Age [months] ^1^	4.69 (0.55)	4.68 (0.56)	4.69 (0.55)	0.909
Sex [% boys]^2^	67.80	71.43	64.52	0.570
Gestational age [weeks]^1^	40.00 (2.00)	40.00 (2.00)	40.00 (1.00)	0.930
Birth weight [g] ^1^	3576.10 (426.28)	3453.21 (445.16)	3687.10 (382.28)	0.034*
Birth length [cm] ^1^	55.29 (2.93)	54.54 (2.99)	55.97 (2.75)	0.060

Group differences were assessed using the t-test or Mann-Whitney U test. Significant differences are asterisked and bolded. ^1^ median, IQR, U-Mann; ^2^ percentage, chi square; BF, body fat; BMI, body mass index; SFA, saturated fatty acid; WHR, waist-to-hip ratio.

### 3.2 Differences in mature milk adipokines and immunoactive factors according to maternal body fat groups

**Figure 1:**
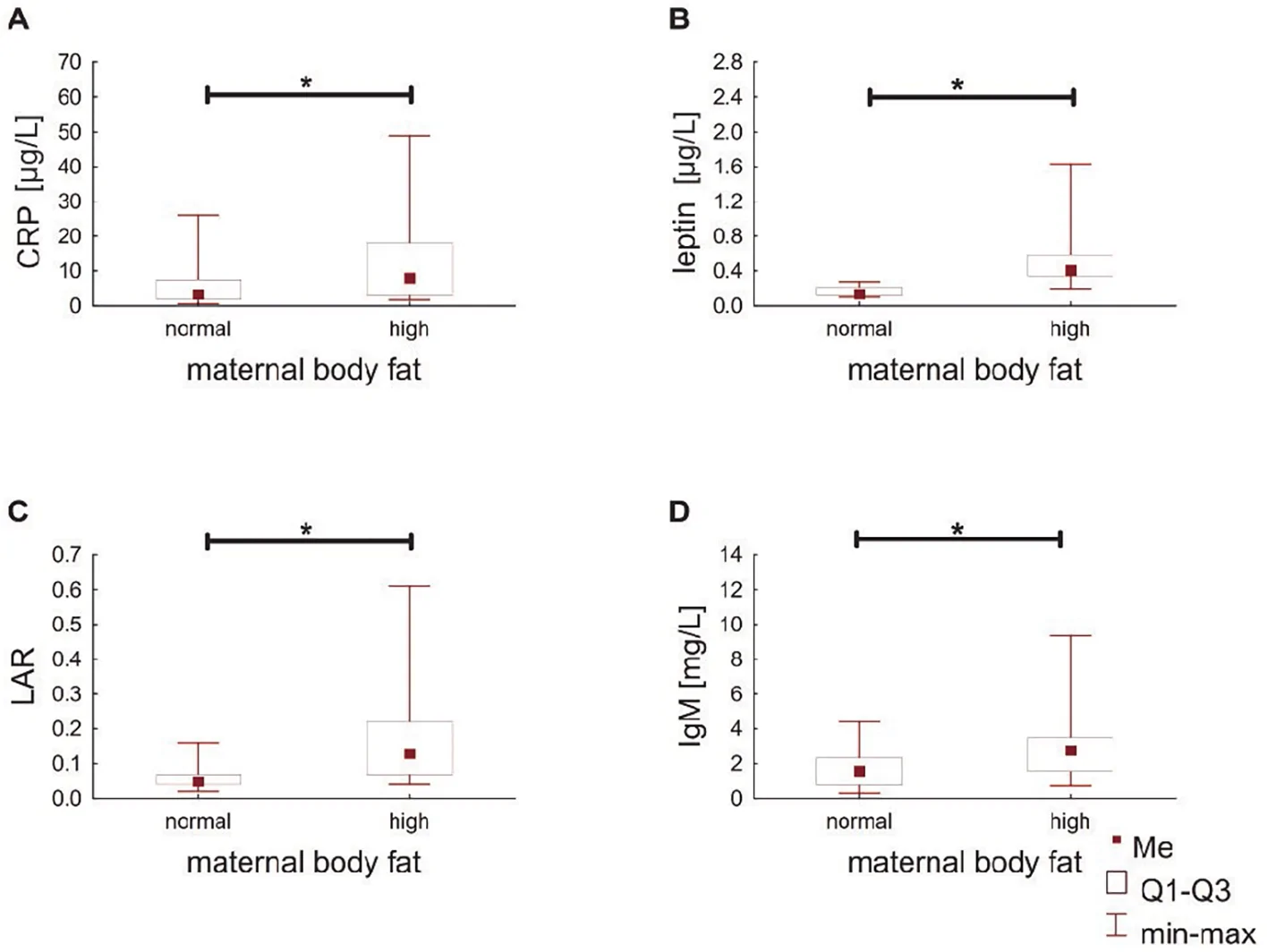
Differences in milk CRP **(A)**, leptin **(B)**, leptin-to-adiponectin ratio [LAR; **(C)**], and IgM **(D)** concentrations between mothers with normal and high body fat (BF). Group differences were assessed using the Mann-Whitney U test. Boxes represent the median (Me) and interquartile range (Q1-Q3), while whiskers indicate minimum and maximum values. The significant differences are asterisked.

Simple comparisons between groups showed significantly higher levels of CRP (8.00 μg/L vs 3.44 μg/L, *p* = 0.005), leptin (0.41 μg/L vs 0.14 μg/L, *p* < 0.001), LAR (0.13 vs 0.05; *p* < 0.001), and IgM (2.78 mg/L vs 1.59 mg/L; *p* = 0.008) in mature milk from mothers with high BF compared with those with normal BF (**Figure 1**). Other examined breast milk components: adiponectin, ghrelin LF, S-IgA, and IgG did not differ significantly between these groups (**Table 2**).

**Table 2:** Results of ANCOVA models for lactoferrin with leptin and LAR as continuous predictors and body fat as a factor.

Predictors	β	*F*_(1,54)_	η^2^	*p*
Model with leptin *F*_(4,54)_=4.20, *p* = 0.005*, R^2adj.^ = 0.18				
BF category [normal]	0.10	2.11	0.04	0.152
Ln leptin	**0.51**	**5.58**	**0.09**	0.022*
Gestational age	**-0.11**	**12.69**	**0.19**	0.001*
SFA intake	0.00	0.02	0.00	0.884
Model with LAR *F*_(4,54)_=3.98, *p* = 0.007*, R^2adj.^ = 0.17				
BF category [normal]	0.06	1.00	0.02	0.322
Ln LAR	**0.37**	**4.84**	**0.08**	0.032*
Gestational age	**-0.11**	**12.18**	**0.18**	0.001*
SFA intake	0.00	0.05	0.00	0.829

The models were adjusted for infant gestational age and maternal SFA intake. Significant differences are asterisked and bolded. BF, body fat; LAR, leptin-to-adiponectin ratio; SFA, saturated fatty acids.

### 3.3 Effect of maternal body fat and adipokines on immunoglobulin and lactoferrin levels in mature milk

**Figure 2:**
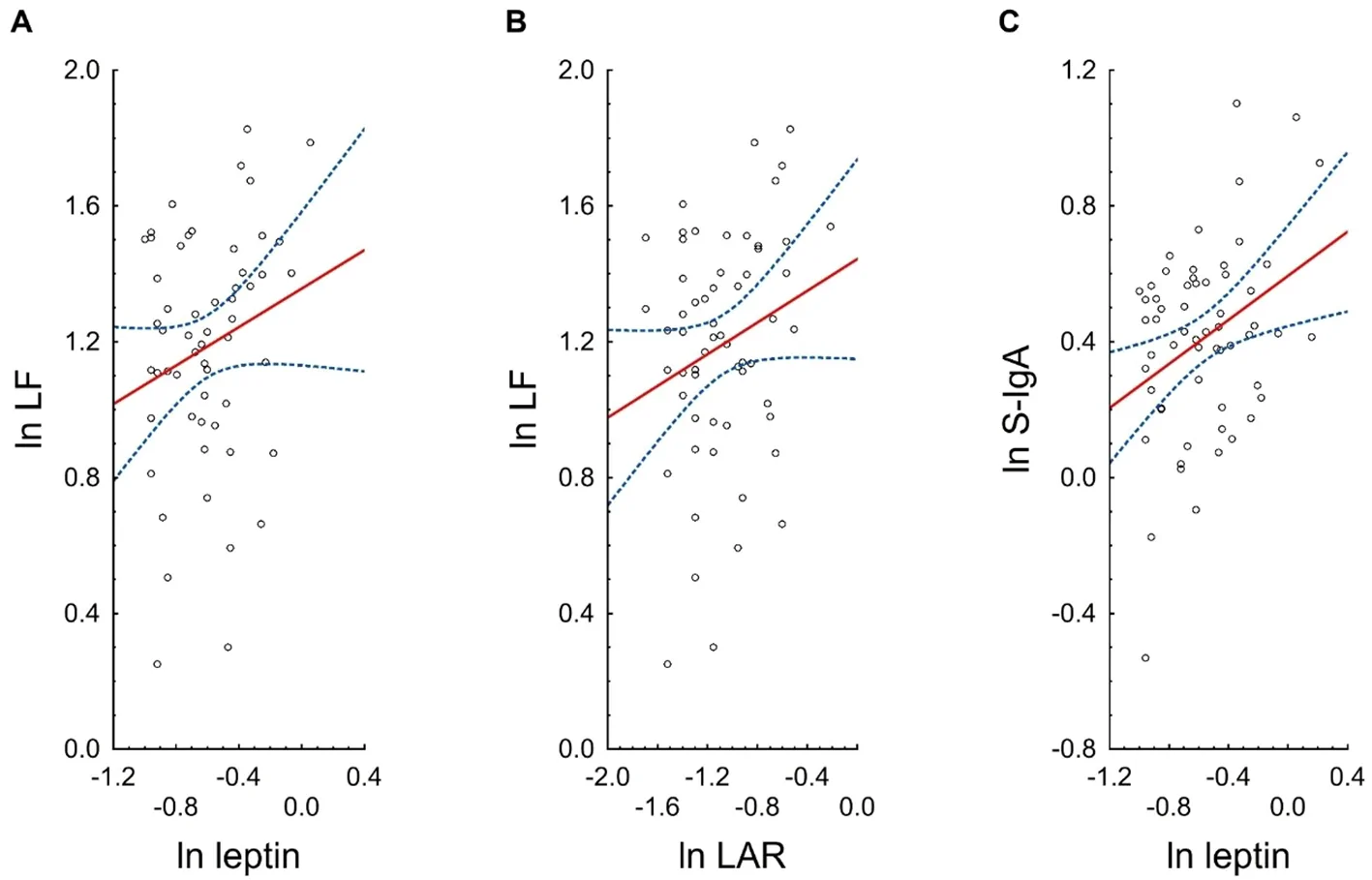
Scatter plots showing associations between lactoferrin (LF) and leptin **(A)**, lactoferrin and the leptin-to-adiponectin ratio (LAR) **(B)**, and secretory immunoglobulin A (S-IgA) and leptin **(C)**. The red line indicates the fitted regression line, whereas the blue dotted lines indicate the 95% confidence limits.

The ANCOVA model for lactoferrin as a dependent variable, including BF group, leptin, gestational age, and maternal intake of SFA, showed a significant positive effect of leptin (β=0.51, *p* = 0.022; **Figure 2**) and gestational age (β =-0.11, *p* = 0.001) on the level of lactoferrin. No significant effect was found for the BF group (*p* = 0.152) and the SFA intake (*p* = 0.884). The whole model was significant (*F*_(4,54)_=4.20, *p* = 0.005, R^2adj.^ = 0.18) and explained 18% of the variance in lactoferrin concentration (**Table 2**).

Similarly, the model for lactoferrin with LAR indicated a positive effect of LAR (β=0.37, *p* = 0.032; **Figure 2**) and a negative effect of gestational age (β=-0.11, *p* = 0.001) on milk lactoferrin. No effect was observed for body fat category (*p* = 0.322) or maternal SFA intake (*p* = 0.829). The whole model was significant (*F*_(4,54)_=3.98, *p* = 0.007, R^2adj.^ = 0.17) and explained 17% of the variance in lactoferrin levels (**Table 2**).

The remaining models for lactoferrin, although significant, indicated the association only with gestational age. Results for these models are presented in **Supplementary Materials** (**Supplementary 3**).

For S-IgA, the models showed a significant positive relationship with leptin (β=0.47, *p* = 0.014; **Figure 2**) and a negative association with gestational age (β=-0.05, *p* = 0.037), but no relationship with body fat category (*p* = 0.239) or SFA intake (*p* = 0.364) (**Table 3**). The whole model for S-IgA, body fat, and leptin was significant (*F*_(4,54)_=3.28, *p* = 0.018, R^2adj.^ = 0.14) and explained 14% of the variance in milk S-IgA levels. The ANCOVA model for S-IgA with LAR did not indicate a significant association (**Table 3**).

**Table 3:** Results of ANCOVA models for S-IgA with leptin and LAR as continuous independent predictors and BF as a factor.

Predictors	B	*F*_(1,54)_	η^2^	*p*
Model with leptin *F*_(4,54)_=3.28, *p* = 0.018*, R^2adj.^ = 0.14				
BF category [normal]	0.07	1.42	0.03	0.239
Ln leptin	**0.47**	**6.41**	**0.11**	0.014*
Gestational age	**-0.05**	**4.59**	**0.08**	0.037*
SFA intake	0.00	0.84	0.02	0.364
Model with LAR *F*_(4,54)_=2.18, *p* = 0.083, R^2adj.^ = 0.08				
BF category [normal]	0.01	0.02	0.00	0.878
Ln LAR	0.23	2.46	0.04	0.123
Gestational age	-0.05	2.71	0.05	0.105
SFA intake	0.00	0.77	0.02	0.385

The models were adjusted for infant gestational age and maternal SFA intake. Significant differences are asterisked and bolded. BF, body fat; LAR, leptin-to-adiponectin ratio; SFA, saturated fatty acids.

The remaining models for S-IgA with other adipokines were not statistically significant (**Supplementary 3**).

The model for IgM with leptin showed no significant relationship. In contrast, the model with LAR indicated only the effect of BF (β=-0.26, *p* = 0.045). No effect was found for LAR (*p* = 0.500), gestational age (*p* = 0.129), and maternal SFA intake (*p* = 0.508). The whole model was significant (*F*_(4.54)_=3.18, *p* = 0.020, R^2adj.^ = 0.13) and explained 13% of the variance in IgM levels (**Table 4**).

**Table 4:** Results of ANCOVA models for IgM with leptin and LAR as continuous independent predictors and BF as a factor.

Predictors	β	*F*(1,54)	η^2^	*p*
Model with leptin *F*_(4,54)_=3.26, *p* = 0.018*, R^2adj.^ = 0.13				
BF category [normal]	-0.22	2.10	0.04	0.153
Ln leptin	0.42	0.73	0.01	0.396
Gestational age	0.11	2.24	0.04	0.140
SFA intake	0.00	0.43	0.01	0.516
Model with LAR *F*_(4,54)_=3.18, *p* = 0.020*, R^2adj.^ = 0.13				
BF category [normal]	**-0.26**	**4.22**	**0.07**	0.045*
Ln LAR	0.25	0.46	0.01	0.500
Gestational age	0.11	2.37	0.04	0.129
SFA intake	0.00	0.44	0.01	0.508

The models were adjusted for infant gestational age and maternal SFA intake. Significant differences are asterisked and bolded. BF, body fat; LAR, leptin-to-adiponectin ratio; SFA, saturated fatty acids.

The remaining IgM models, although significant, showed an association only with the maternal BF category. Results for these models are presented in the **Supplementary Materials** (**Supplementary 5**).

### 3.4 Effect of maternal WHR and adipokines on immunoglobulin and lactoferrin levels in mature milk

The ANCOVA model for S-IgA as the dependent variable, including WHR group, leptin, gestational age, and maternal SFA intake, showed a significant positive effect of leptin (β=0.33, *p* = 0.018; **Table 5**). No significant effects were found for the WHR group (*p* = 0.760) or other covariates. The overall model was significant (*F*_(4,54)_=2.89, *p* = 0.031, R^2adj.^ = 0.12), accounting for 12% of the variance in S-IgA concentration. However, the model for S-IgA with LAR did not show a significant association. Similarly, no significant differences were observed in the models for S-IgA with adiponectin or ghrelin (**Supplementary 8**).

**Table 5:** Results of ANCOVA models for S-IgA with leptin and LAR as continuous independent predictors and WHR as a factor.

Predictors	β	*F*(1,54)	η^2^	*p*
Model with leptin *F*_(4,54)_=2.89, *p* = 0.031*, R^2adj.^ = 0.12				
WHR category [normal]	0.01	0.09	0.00	0.760
Ln leptin	0.33	5.93	0.10	0.018*
Gestational age	-0.05	3.00	0.05	0.089
SFA intake	0.00	0.53	0.01	0.470
Model with LAR *F*_(4,54)_=2.21, *p* = 0.080, R^2adj.^ = 0.08				
WHR category [normal]	0.02	0.13	0.00	0.716
Ln LAR	0.25	3.45	0.06	0.069
Gestational age	-0.05	2.99	0.05	0.090
SFA intake	0.00	0.70	0.01	0.405

The models were adjusted for infant gestational age and maternal SFA intake. Significant differences are asterisked and bolded. LAR, leptin-to-adiponectin ratio; SFA, saturated fatty acids; WHR, waist-to-hip ratio.

Interestingly, the model for IgM with WHR category and leptin indicates that leptin was positively associated with IgM (β=1.01, *p* = 0.006; **Table 6**). The other independent variables, including WHR category, were not significantly associated. The full model was significant (*F*_(4,54)_=2.68, *p* = 0.042, R^2adj.^ = 0.11), explaining 11% of the variance in IgM concentration. Models for other adipokines were not significant (**Supplementary 10**).

**Table 6:** Results of ANCOVA models for IgM with leptin and LAR as continuous independent predictors and WHR as a factor.

Predictors	β	*F*_(1,54)_	η^2^	*p*
Model with leptin *F*_(4,54)_=2.68, *p* = 0.042*, R^2adj.^ = 0.11				
WHR category [normal]	0.08	0.56	0.01	0.458
Ln leptin	**1.01**	**8.06**	**0.13**	0.006*
Gestational age	0.10	1.77	0.03	0.189
SFA intake	0.00	0.19	0.00	0.662
Model with LAR *F*_(4,54)_=2.12, *p* = 0.091, R^2adj.^ = 0.07				
WHR category [normal]	0.10	0.84	0.02	0.363
Ln LAR	0.85	5.91	0.10	0.018*
Gestational age	0.09	1.47	0.03	0.231
SFA intake	0.00	0.12	0.00	0.730

The models were adjusted for infant gestational age and maternal SFA intake. Significant differences are asterisked and bolded. LAR, leptin-to-adiponectin ratio; SFA, saturated fatty acids; WHR, waist-to-hip ratio.

None of the models for LF and IgG with the WHR category were significant (**Supplementary 7**, **9**).

## 4 Discussion

In this study, we hypothesized that increased maternal BF accumulation is associated with altered composition of mature human milk, thereby affecting levels of inflammatory, immunological, and metabolic biomarkers. In line with our hypothesis, comparisons between mothers in high- and normal-BF groups showed that higher maternal BF was associated with higher levels of mature milk CRP and leptin, as well as the leptin-to-adiponectin ratio. Furthermore, higher milk leptin was associated with higher levels of milk S-IgA and lactoferrin. A similar effect on milk lactoferrin was observed with the leptin-to-adiponectin ratio. Contrary to our hypothesis, mature milk adiponectin levels were not associated with milk IgG or IgM levels, although milk IgM levels differed between mothers with higher and normal BF in the models. Additionally, milk IgG levels did not differ between BF groups and were not associated with milk adiponectin levels. We did not observe any association between WHR and milk immunoactive factors.

We focused on BF because it provides a more direct estimate of maternal adiposity and body composition than BMI. Because adipose tissue is the primary source of adipokines, which may influence immunoglobulin and CRP levels in breast milk, we considered BF a more accurate anthropometric parameter for evaluating the adipokine-immunoglobulin-CRP interplay in human milk. Overweight and obesity are characterized by low-grade chronic inflammation and altered immune responses. Among inflammatory markers, CRP is one of the most widely measured indicators of low-grade inflammation in obstetric settings. Serum and milk CRP levels are strongly associated with overweight and obesity, maternal metabolic dysfunction, and adverse child outcomes (100–102). Results of our analysis indicating higher milk CRP concentrations in the high BF group corroborate previous reports showing that maternal pre-pregnancy overweight/obesity, defined by BMI and/or excessive weight gain, is associated with significantly elevated milk CRP concentrations (77, 103). Whitaker and coworkers (77) reported that milk CRP concentration, ranging from 8.0 to 43.9 μg/L, decreased during the third month of lactation. Also, its level was higher in mothers with overweight/obesity and higher gestational weight gain (GWG). Specifically, normal-weight women exceeding GWG guidelines had significantly lower CRP concentrations than women with obesity, regardless of GWG category. Also, women with obesity experiencing excessive GWG had significantly higher CRP concentrations than all other groups (77). Thus, the increased CRP concentration in mature milk observed in our study indicates ongoing low-grade inflammation associated with high BF accumulation observed in obesity.

Among a variety of metabolic biomarkers, most research focuses on the associations between obesity and leptin and adiponectin levels. The presence of adipokines in human milk results from two distinct mechanisms: *de novo* synthesis by mammary gland epithelial cells and transfer from the maternal circulation following their secretion from white adipose tissue into the bloodstream (104–106). The content of adipokines in specific pathways was not defined. Higher maternal adiposity has consistently been associated with higher milk leptin concentrations in both colostrum and mature milk collected between the 3rd and 6th months of lactation (37, 107–112). Chan et al. (113) similarly reported a strong association between maternal BMI and milk leptin, but not milk adiponectin. Consistent with this literature, we observed significantly higher leptin concentrations in milk from mothers with high BF than in milk from those with normal BF. In contrast, milk adiponectin concentrations did not differ significantly between groups. However, we observed a weak negative association between milk adiponectin and maternal waist-to-hip ratio (see **Supplementary Materials**), suggesting that central adiposity may be more closely related to milk adiponectin than overall body fat.

The lower mature milk adiponectin levels in mothers with higher WHR are consistent with the plasma adiponectin decline observed in obesity, which is driven by chronic low-grade inflammation and insulin resistance (42). The leptin-to-adiponectin ratio, a circulating biomarker of adipose tissue dysfunction (114), was significantly higher in the mature milk of mothers with high BF than in that of those with normal BF. To our knowledge, this is the first report of such an association in mature human milk. The lack of an effect of high maternal BF on milk ghrelin observed in our study aligns with the findings of Andreas et al. (37). These authors also reported no association between ghrelin concentration in mature human milk at 1 week and 3 months and maternal BMI. In contrast, Tekin Guler et al. (115) demonstrated that ghrelin concentration in breast milk at the 2nd month of lactation changes between pre-feed and post-feed human milk, but remains elevated in milk collected from mothers with obesity. Although data on milk ghrelin remain inconsistent, our results generally support the concept that maternal adiposity signals, particularly circulating leptin, are transmitted to human milk and may contribute to shaping infant appetite regulation and the offspring growth trajectory.

Evidence on the association between maternal adiposity and milk IgM remains limited and inconsistent. Dyndar et al. (116) reported a 1.95-fold reduction in transitional milk IgM concentrations among women with class III obesity, whereas no differences were observed in class I or II obesity compared with normal-weight mothers. In contrast, we found that mothers with high BF had significantly higher IgM concentrations in mature milk than those with normal BF. Differences in lactation stage, adiposity assessment, and the severity of maternal obesity may contribute to these discrepant findings.

We hypothesized that higher body fat may be associated with changes in class-switch recombination in B cells, which could potentially shift the balance between IgM and switched isotypes and, in turn, may influence the recruitment of IgM-producing B cells to the mammary gland. Our observation of higher IgM concentrations in the milk of mothers with higher BF is consistent with this hypothesis, although causal links remain speculative. We further hypothesize that increased IgM levels might reflect activation of protective IgM-mediated pathways described in adipose tissue B-1 cells, which have been shown to limit inflammation and metabolic dysfunction in human and mouse models of systemic molecular events (95, 117, 118).

Although values for milk IgG in our study align with data from previous work (22, 119) we found no difference in mature milk IgG and S-IgA between mothers with normal and high BF. This may partly reflect the relatively modest adiposity in our cohort: the median maternal BMI was 26.48 kg/m², which falls within the overweight rather than obese range. By contrast, Dyndar et al. (116) reported lower total IgA concentrations in transitional milk from mothers with class II (1.56-fold) and class III (2.96-fold) obesity, as well as lower IgG concentrations in women with class III obesity. Such reductions could weaken the passive immune protection provided by milk against gastrointestinal and respiratory infections, although they may also reflect obesity-related alterations in maternal immune function (50). Direct comparison between our findings and those of Dyndar et al. (116) is limited by important methodological differences. These include the assessment of maternal adiposity (BMI versus body fat content), the stage of lactation studied (second week versus fifth month), and the immunoglobulin measures used (total IgA versus S-IgA). Therefore, we suggest that the marked immunoglobulin differences reported by Dyndar et al. may be driven primarily by the inclusion of mothers with severe, class III obesity rather than by elevated adiposity across the full range of maternal body composition. This study presents the first integrated analysis of how BF accumulation affects both the metabolic and immunological components of mature maternal milk. We use breast milk samples collected in the 5th month of lactation, when milk composition is relatively stable and considered mature, allowing a more reliable assessment of adipokine and immunoglobulin profiles. This timing also minimizes the influence of early postpartum physiological variability, which may persist until 18 weeks postpartum (120–122). Thus, sampling at 5 months postpartum reflects stabilized milk composition and advanced maternal recovery (84, 120–122). Although no significant differences in LF and S-IgA concentrations were observed among BF groups, our study revealed significant associations between mature milk LF and leptin and LAR, as well as between S-IgA and leptin. These results suggest that increased maternal fat accumulation may disrupt her metabolic profile, leading to higher plasma leptin levels, which translate into increased milk leptin and a higher leptin-to-adiponectin ratio, and to altered milk immunoactive factors.

The associations observed in our study resemble previously described leptin effects mediated through the leptin receptor in regulating innate and adaptive immune responses (123, 124). This study presents an association between human milk leptin and S-IgA, indicating coordinated regulation of metabolic and immune components within the gut-mammary axis. Niimi et al. (125) reported that recruitment of IgA-producing plasma cells into the mammary gland and the transport of secreted IgA into mammary alveoli are controlled endogenously. Thus, the interplay between leptin and S-IgA observed in our study may reflect gut-adipose immunometabolic crosstalk, functionally linking leptin concentrations with mucosal immune activity. Secretory Immunoglobulin A production in mucosal tissues occurs via two distinct pathways: T cell-dependent (TD) and T cell-independent (TI). High leptin levels are well documented to inhibit the proliferation and function of certain immune cell subsets, including regulatory T cells, which are important for the TD differentiation of S-IgA-secreting B cells (123, 126). Thus, our results may suggest that T cell-dependent mechanisms are partially inhibited in a hyperleptinemic milieu, whereas T cell-independent pathways could continue to support or compensate for IgA production. This dual-pathway framework provides a plausible explanation for how leptin exposure during early life may differentially shape the maturation of mucosal immunity. Moreover, in an animal model, leptin supplementation during the suckling period reduced intestinal S-IgA concentration; however, a trophic effect on the developing rat intestine was observed (127). The authors suggested that leptin and adiponectin during suckling contribute to the maturation of mucosal immunity in early life in rats. These findings support the notion that leptin-driven modulation of TD and TI pathways, together with adipokine-mediated trophic effects, may underlie the complex relationship among maternal adiposity, milk leptin, and the developing mucosal immune system of the offspring.

The observed associations between mature human milk lactoferrin and leptin may also reflect a shared response to obesity-related disturbances in adipose tissue iron homeostasis, oxidative stress, and low-grade inflammation, which influence both adipokine secretion and iron-binding protein dynamics in the mammary gland. Higher maternal body fat content, associated with systemic and local inflammation, increased vascular and epithelial permeability, together with neutrophil infiltration, may promote ‘leakage’ of iron and neutrophil-derived lactoferrin into the mammary gland and, consequently, into human milk. In this context, elevated leptin levels, together with lactoferrin, could indicate a coordinated immunometabolic adaptation to disrupted iron distribution and subclinical inflammation in mothers with higher body fat. The observed associations suggest that adiposity-driven increases in leptin may enhance immune defense as a compensatory response to the metabolic and inflammatory stress associated with abnormal fat accumulation. The mechanisms underlying the higher LF/S-IgA concentration in response to increased leptin levels remain unknown. To the best of our knowledge, no studies have examined these associations to date. Leptin, acting beyond appetite regulation, influences immune cell function and mucosal immunity (123), which could explain its associations with S-IgA and lactoferrin. This coordinated increase in milk leptin and S-IgA supports neonatal gut barrier maturation, pathogen neutralization, and mucosal immunity.

The observed positive association between leptin and lactoferrin concentrations suggests that maternal abnormal fat accumulation, typically associated with low-grade inflammation, may contribute to higher LF levels as a potential compensatory mechanism to mitigate metabolic stress. Maternal metabolic status modulates LF through an interplay of inflammation, hormones, and nutrition. Jamka et al. (128) found no differences in LF between metabolically healthy women with obesity and metabolically unhealthy women with obesity. Meanwhile, Sindi et al. (129) reported that human milk lactoferrin levels decreased by approximately 20.5% over two weeks after dietary modification among mothers exclusively breastfeeding term infants aged 1.6 to 4.9 months. The findings of this study underscore the potential for intergenerational immunometabolic programming mediated by maternal milk, as previously reported by Erliana and Fly (130) and by Arenas et al. (131).

In our study, we did not find associations between maternal SFA intake and adipokines or immune components in mature human milk. To date, studies have shown that human milk from mothers with overweigth/obesity contains higher levels of SFA (132–136). Santos et al. (137) reviewed the impact of SFA on inflammation and circulating levels of adipokines, suggesting a potential positive association of SFA with CRP but not with adipokines. Harvey et al. (138) reported that SFAs can promote systemic inflammation, whereas Maramulla et al. (139) found that SFAs and PUFAs (polyunsaturated fatty acids) were not proinflammatory in human adipose tissue and adipocyte models. Taken together, these findings suggest that adverse inflammatory effects attributed to dietary SFA often co-occur with obesity and broader dietary or lifestyle changes, making it difficult to isolate the specific contribution of SFA alone. In view of the above, the lack of correlation between maternal SFA consumption, milk adipokines, and immune factors in our study subjects is consistent with the current literature, indicating that, at least within the observed range of maternal SFA consumption, SFA plays a minor role in determining the profile of adipokines and immunoglobulins in mature human milk.

Our study uniquely evaluates immune and metabolic milk components at the 5th month of lactation, a period that likely reduces physiological variability and enables a more reliable assessment of adipokine concentrations, thereby minimizing confounding effects of dynamic changes in early lactation. The methodological design of this study uses a well-characterized maternal cohort. Excluding mothers with severe metabolic conditions and infections that could confound the metabolic and immune profiles of human milk enabled the isolation of adiposity-specific effects. The cohort’s homogeneity in location and ethnic group minimized geographic influences on the metabolic and immunological components of human milk (140, 141). Nevertheless, several limitations should be acknowledged. Although the study was conducted in a carefully characterized and relatively homogeneous cohort, the small sample size and specific characteristics of the study population may limit the generalizability of our findings. Therefore, our findings should be interpreted with caution and confirmed in larger, independent cohorts.

The study group consisted of mothers with higher socioeconomic status and higher levels of education. These characteristics might affect maternal dietary choices and health behaviors, which in turn may influence maternal immunometabolic status and adiposity. Thus, future studies should include mothers from more diverse socioeconomic and educational groups. Moreover, due to the limited number of participants, we were able to control the analysis for only a few selected, statistically significant factors (gestational age, body fat group, and selected fatty acid intake). Interpretation might also be constrained by unmeasured factors, such as gestational weight gain, which influence leptin concentrations in mature milk at 3–4 months of lactation (142). Larger prospective studies with structured follow-up are needed to evaluate how maternal adiposity affects the metabolic and immunological components of human milk and their links to early offspring metabolic programming.

## 5 Conclusion

This study presents the first integrated analysis of metabolic and immunological components in mature milk from mothers with overweight or obesity, demonstrating that elevated leptin levels are associated with higher concentrations of immunoactive factors, such as S-IgA and LF. Our findings provide novel insights into the bioactive properties of maternal milk in the context of early immunometabolic programming and highlight their potential relevance for the health trajectories of infants born to mothers with increased fatness, with possible long-termimplications for offspring growth and disease risk. These findings reveal potential intergenerational metabolic and immunological programming via altered milk bioactives. Future research should incorporate lactation stage, maternal diet, and inflammatory markers to resolve inter-study inconsistencies.

## Data Availability

The dataset analyzed in this study is publicly available in the RepOD repository at doi:10.18150/6WKIZ1 (143). The dataset is licensed under the Creative Commons Attribution (CC BY) license.
