# Supplementary material for: Adiposity-dependent adipokine-immunoglobulin interplay in mature breast milk
Source: Front Endocrinol (Lausanne). 2026 Aug 28;17:1873967. doi: 10.3389/fendo.2026.1873967 (PMC13561822; doi:10.3389/fendo.2026.1873967)
Supplement: Supplementary file 1 [file Table1.docx]

**Supplementary materials**

Supplementary 1. Characteristics of milk parameters in a whole sample and groups of mothers with normal and high BF (U-Mann-Whitney test).

|  | All  N=59 | Normal BF  N=28 | High BF  N=31 | *p* |
| --- | --- | --- | --- | --- |
| Milk CRP [μg/L] | 4.50 (12.00) | 3.44 (5.69) | 8.00 (15.00) | **0.005*** |
| Milk leptin [μg/L] | 0.25 (0.28) | 0.14 (0.10) | 0.41 (0.25) | **<0.001*** |
| Milk adiponectin [μg/L] | 3.17 (2.54) | 3.17 (2.15) | 3.17 (2.88) | 0.851 |
| Milk LAR** | 0.07 (0.10) | 0.05 (0.02) | 0.13 (0.15) | **<0.001*** |
| Milk ghrelin [μg/L] | 0.42 (0.11) | 0.44 (0.09) | 0.42 (0.13) | 0.712 |
| Milk LF [g/L] | 3.42 (1.70) | 3.34 (1.57) | 3.55 (1.60) | 0.338 |
| Milk S-IgA [g/L] | 1.54 (0.51) | 1.55 (0.52) | 1.54 (0.51) | 0.361 |
| Milk IgG [mg/L] | 10.67 (3.24) | 10.27 (2.71) | 10.91 (4.47) | 0.757 |
| Milk IgM [mg/L] | 2.12 (2.16) | 1.59 (1.57) | 2.78 (1.918) | **0.008*** |

The significant differences are asterisked. **LAR - leptin-to-adiponectin ratio; BF - body fat; LF- lactoferrin; CRP - C-reactive protein

Supplementary 2. Correlation matrix.

|  | Breast index | WHR | Ln LF | Ln S-IgA | Ln IgG | Ln IgM | Ln CRP | Ln leptin | Ln adiponectin | Ln LAR | Ln ghrelin |
| --- | --- | --- | --- | --- | --- | --- | --- | --- | --- | --- | --- |
| Breast index |  | r=-0.14  *p*=0.306 | r=-0.01  *p*=0.922 | r=0.23  *p*=0.077 | r=0.08  *p*=0.567 | r=0.01  *p*=0.953 | r=0.01  *p*=0.969 | r=0.08  *p*=0.559 | **r=0.32**  ***p*=0.014*** | r=-0.14  *p*=0.280 | r=0.06  *p*=0.635 |
| WHR |  |  | r=0.25  *p*=0.057 | r=0.20  *p*=0.138 | r=0.14  *p*=0.290 | r=0.08  *p*=0.530 | r=0.15  *p*=0.253 | **r=0.39**  ***p*=0.003*** | r=-0.26  *p*=0.051 | **r=0.52**  ***p*<0.001*** | r=0.15  *p*=0.277 |
| Ln LF |  |  |  | **r=0.42**  ***p*=0.001*** | r=0.10  *p*=0.456 | r=0.01  *p*=0.937 | r=0.07  *p*=0.602 | r=0.21  *p*=0.107 | r=0.03  *p*=0.844 | r=0.20  *p*=0.131 | r=0.21  *p*=0.107 |
| Ln S-IgA |  |  |  |  | r=0.18  *p*=0.166 | **r=0.31**  ***p*=0.015*** | r=0.10  *p*=0.434 | r=0.23  *p*=0.078 | r=0.09  *p*=0.507 | r=0.15  *p*=0.268 | r=0.15  *p*=0.245 |
| Ln IgG |  |  |  |  |  | **r=0.26**  ***p*=0.043*** | r=-0.05  *p*=0.722 | r=0.19  *p*=0.159 | r=0.18  *p*=0.173 | r=0.04  *p*=0.739 | r=-0.26  *p*=0.050 |
| Ln IgM |  |  |  |  |  |  | r=0.16  *p*=0.236 | r=0.34  *p*=0.009 | r=-0.02  *p*=0.871 | r=0.35  *p*=0.006 | r=0.04  *p*=0.758 |
| Ln CRP |  |  |  |  |  |  |  | **r=0.36**  ***p*=0.004*** | r=-0.13  *p*=0.324 | **r=0.44**  ***p*<0.001*** | r=-0.01  *p*=0.915 |
| Ln leptin |  |  |  |  |  |  |  |  | r=0.10  *p*=0.434 | **r=0.77**  ***p*<0.001*** | r=-0.08  *p*=0.563 |
| Ln adiponectin |  |  |  |  |  |  |  |  |  | **r=-0.53**  ***p*<0.001*** | r=-0.16  *p*=0.216 |
| Ln LAR |  |  |  |  |  |  |  |  |  |  | r=0.06  *p*=0.674 |

*Bold values represent correlations with statistical significance (*p* < 0.05). CRP - C-reactive protein; LF - lactoferrin; LAR – leptin-to-adiponectin ratio; WHR – waist-to-hip ratio

Supplementary 3. The ANCOVA model results for lactoferrin.

|  | β | *F*_(1,54)_ | η^2^ | *p* |
| --- | --- | --- | --- | --- |
| **Model with adiponectin** *F*_(4,54)_=2.56, *p*=**0.049***, R^2^_adj._=0.10 | | | | |
| BF category [normal] | -0.02 | 0.28 | 0.01 | 0.601 |
| Ln adiponectin | -0.05 | 0.07 | 0.00 | 0.786 |
| Gestational age | -0.08 | 9.10 | 0.14 | **0.004*** |
| Saturated fatty acids intake | 0.00 | 0.00 | 0.00 | 0.977 |
| **Model with ghrelin** *F*_(4,54)_=2.94, *p*=**0.029***, R^2^_adj._=0.12 | | | | |
| BF category [normal] | -0.03 | 0.49 | 0.01 | 0.486 |
| Ln ghrelin | 0.54 | 1.34 | 0.02 | 0.252 |
| Gestational age | -0.09 | 8.03 | 0.13 | **0.006*** |
| SFA intake | 0.00 | 0.03 | 0.00 | 0.856 |

The model was adjusted for gestational age and saturated fatty acids. The significant associations are asterisked. BF - body fat; SFA - saturated fatty acids

Supplementary 4. The ANCOVA model results for S-IgA.

|  | β | *F*_(1,54)_ | η^2^ | *p* |
| --- | --- | --- | --- | --- |
| **Model with adiponectin** *F*_(4,54)_=1.54, *p*=0.203, R^2^_adj._=0.10 | | | | |
| BF category [normal] | -0.04 | 1.13 | 0.02 | 0.292 |
| Ln adiponectin | 0.06 | 0.14 | 0.00 | 0.712 |
| Gestational age | -004 | 1.76 | 0.03 | 0.190 |
| SFA intake | 0.00 | 1.05 | 0.02 | 0.309 |
| **Model with ghrelin** *F*_(4,54)_=1.90, *p*=0.124, R^2^_adj._=0.06 | | | | |
| BF category [normal] | -0.05 | 1.64 | 0.03 | 0.206 |
| Ln ghrelin | 0.47 | 1.43 | 0.03 | 0.237 |
| Gestational age | -0.03 | 1.38 | 0.02 | 0.246 |
| SFA intake | 0.00 | 0.69 | 0.01 | 0.411 |

The model was adjusted for gestational age and saturated fatty acids. The significant associations are asterisked. BF - body fat; SFA - saturated fatty acids

Supplementary 5. The ANCOVA model results for IgM.

|  | β | *F*_(1,54)_ | η^2^ | *p* |
| --- | --- | --- | --- | --- |
| **Model with adiponectin** *F*_(4,54)_=2.70, *p*=**0.040***, R^2^_adj._=0.10 | | | | |
| BF category [normal] | -0.73 | 8.89 | 0.14 | **0.004*** |
| Ln adiponectin | 0.46 | 0.19 | 0.00 | 0.664 |
| Gestational age | 0.29 | 2.70 | 0.05 | 0.106 |
| SFA intake | -0.01 | 0.52 | 0.01 | 0.474 |
| **Model with ghrelin** *F*_(4,54)_=2.76, *p*=**0.037***, R^2^_adj._=0.11 | | | | |
| BF category [normal] | -0.76 | 9.48 | 0.15 | **0.003*** |
| Ln ghrelin | 1.62 | 0.41 | 0.01 | 0.526 |
| Gestational age | 0.30 | 2.89 | 0.05 | 0.096 |
| SFA intake | -0.01 | 0.70 | 0.01 | 0.406 |

The model was adjusted for gestational age and saturated fatty acids. The significant associations are asterisked. BF-body fat; SFA - saturated fatty acids

Supplementary 6. The ANCOVA model results for IgG.

|  | β | *F*_(1,54)_ | η^2^ | *p* |
| --- | --- | --- | --- | --- |
| **Model with leptin** *F*_(4,54)_=1.43, *p*=0.237, R^2^_adj._=0.03 | | | | |
| BF category [normal ] | 0.06 | 0.95 | 0.02 | 0.335 |
| Ln leptin | 0.36 | 1.03 | 0.02 | 0.314 |
| Gestational age | 0.03 | 3.24 | 0.06 | 0.078 |
| SFA intake | 0.00 | 0.44 | 0.01 | 0.512 |
| **Model with LAR** *F*_(4,54)_=0.59, *p*=0.674, R^2^_adj._=-0.03 | | | | |
| BF category [normal] | -0.03 | 0.26 | 0.00 | 0.614 |
| Ln LAR | -0.01 | 0.01 | 0.00 | 0.941 |
| Gestational age | 0.04 | 1.83 | 0.03 | 0.182 |
| SFA intake | 0.00 | 0.28 | 0.01 | 0.597 |
| **Model with adiponectin** *F*_(4,54)_=1.31, *p*=0.278, R^2^_adj._=0.02 | | | | |
| BF category [normal] | -0.02 | 0.28 | 0.01 | 0.600 |
| Ln adiponectin | 0.28 | 2.78 | 0.05 | 0.101 |
| Gestational age | 0.04 | 2.22 | 0.04 | 0.142 |
| SFA intake | 0.00 | 0.20 | 0.00 | 0.658 |
| **Model with ghrelin** *F*_(4,54)_=1.52, *p*=0.208, R^2^_adj._=0.03 | | | | |
| BF category [normal] | -0.01 | 0.09 | 0.00 | 0.767 |
| Ln ghrelin | -0.78 | 3.60 | 0.06 | 0.063 |
| Gestational age | 0.03 | 1.22 | 0.02 | 0.274 |
| SFA intake | 0.00 | 0.07 | 0.00 | 0.787 |

The model was adjusted for gestational age and saturated fatty acids. The significant associations are asterisked. BF - body fat; SFA - saturated fatty acids, LAR – leptin-to-adiponectin ratio

Supplementary 7. The ANCOVA model results for lactoferrin.

|  | β | *F*_(1,54)_ | η^2^ | *p* |
| --- | --- | --- | --- | --- |
| **Model with leptin** *F*_(4,54)_=3.12, *p*=**0.022***, R^2^_adj._=0.13 | | | | |
| WHR category [normal] | 0.00 | 0.00 | 0.00 | 0.981 |
| Ln leptin | 0.27 | 2.76 | 0.05 | 0.103 |
| Gestational age | -0.10 | 9.59 | 0.15 | **0.003*** |
| SFA intake | 0.00 | 0.13 | 0.00 | 0.717 |
| **Model with LAR** *F*_(4,54)_=3.26, *p*=**0.018***, R^2^_adj._=0.14 | | | | |
| WHR category [normal] | 0.01 | 0.09 | 0.00 | 0.765 |
| Ln LAR | 0.27 | 3.26 | 0.06 | 0.077 |
| Gestational age | -0.10 | 10.19 | 0.16 | **0.002*** |
| SFA intake | 0.00 | 0.16 | 0.00 | 0.689 |
| **Model with adiponectin** *F*_(4,54)_=2.31, *p*=0.070, R^2^_adj._=0.08 | | | | |
| WHR category [normal] | -0.03 | 0.479 | 0.01 | 0.492 |
| Ln adiponectin | -0.01 | 0.00 | 0.00 | 0.957 |
| Gestational age | -0.09 | 8.31 | 0.16 | **0.006*** |
| SFA intake | 0.00 | 0.00 | 0.00 | 0.962 |
| **Model with ghrelin** *F*_(4,54)_=2.60, *p*=**0.046***, R^2^_adj._=0.10 | | | | |
| WHR category [normal] | -0.03 | 0.38 | 0.01 | 0.543 |
| Ln ghrelin | 0.47 | 1.02 | 0.02 | 0.316 |
| Gestational age | -0.09 | 7.57 | 0.12 | **0.008*** |
| SFA intake | 0.00 | 0.00 | 0.00 | 0964 |

The model was adjusted for gestational age and saturated fatty acids. The significant associations are asterisked. LAR – leptin-to-adiponectin ratio; SFA - saturated fatty acids; WHR – waist-to-hip ratio

Supplementary 8. The ANCOVA model results for S-IgA.

|  | β | *F*_(1,54)_ | η^2^ | *p* |
| --- | --- | --- | --- | --- |
| **Model with adiponectin** *F*_(4,54)_=1.36, *p*=0.261, R^2^_adj._=0.02 | | | | |
| WHR category [normal] | -0.03 | 0.64 | 0.01 | 0.429 |
| Ln adiponectin | 0.10 | 0.34 | 0.01 | 0.560 |
| Gestational age | -0.04 | 1.95 | 0.04 | 0.168 |
| SFA intake | 0.00 | 1.75 | 0.03 | 0.191 |
| **Model with ghrelin** *F*_(4,54)_=1.49, *p*=0.219, R^2^_adj._=0.03 | | | | |
| WHR category [normal] | -0.02 | 0.33 | 0.00 | 0.565 |
| Ln ghrelin | 0.36 | 0.82 | 0.02 | 0.370 |
| Gestational age | -0.04 | 1.74 | 0.03 | 0.192 |
| SFA intake | 0.00 | 1.50 | 0.03 | 0.226 |

The model was adjusted for gestational age and saturated fatty acids. The significant associations are asterisked. SFA - saturated fatty acids; WHR – waist-to-hip ratio

Supplementary 9. The ANCOVA model results for IgG.

|  | β | *F*_(1,54)_ | η^2^ | *p* |
| --- | --- | --- | --- | --- |
| **Model with leptin** *F*_(4,54)_=0.02, *p*=0.307, R^2^_adj._=0.02 | | | | |
| WHR category [normal] | -0.01 | 0.07 | 0.00 | 0.792 |
| Ln leptin | 0.17 | 1.43 | 0.03 | 0.237 |
| Gestational age | 0.04 | 2.20 | 0.04 | 0.144 |
| SFA intake | 0.00 | 0.66 | 0.01 | 0.419 |
| **Model with LAR** *F*_(4,54)_=0.89, *p*=0.474, R^2^_adj._=-0.01 | | | | |
| WHR category [normal] | -0.04 | 0.76 | 0.01 | 0.386 |
| Ln LAR | -0.05 | 0.14 | 0.00 | 0.709 |
| Gestational age | 0.05 | 2.59 | 0.05 | 0.113 |
| SFA intake | 0.00 | 0.17 | 0.00 | 0.678 |
| **Model with adiponectin** *F*_(4,54)_=1.98, *p*=0.111, R^2^_adj._=0.06 | | | | |
| WHR category [normal] | -0.05 | 1.62 | 0.03 | 0.209 |
| Ln adiponectin | 0.35 | 4.21 | 0.07 | **0.045*** |
| Gestational age | 0.05 | 3.23 | 0.06 | 0.078 |
| SFA intake | 0.00 | 0.25 | 0.00 | 0.622 |
| **Model with ghrelin** *F*_(4,54)_=1.94, *p*=0.117, R^2^_adj._=0.06 | | | | |
| WHR category [normal] | -0.04 | 1.09 | 0.02 | 0.301 |
| Ln ghrelin | -0.82 | 4.09 | 0.07 | **0.048*** |
| Gestational age | 0.04 | 1.80 | 0.03 | 0.185 |
| SFA intake | 0.00 | 0.13 | 0.00 | 0.724 |

The model was adjusted for gestational age and saturated fatty acids. The significant associations are asterisked. BF - body fat; LAR – leptin-to-adiponectin ratio; SFA - saturated fatty acids; WHR – waist-to-hip ratio

Supplementary 10. The ANCOVA model results for IgM.

| IgM | β | *F*_(1,54)_ | η^2^ | *p* |
| --- | --- | --- | --- | --- |
| **Model with adiponectin** *F*_(4,54)_=0.62, *p*=0.650, R^2^_adj._=-0.03 | | | | |
| WHR category [normal] | -0.05 | 0.22 | 0.00 | 0.643 |
| Ln adiponectin | 0.19 | 0.17 | 0.00 | 0.683 |
| Gestational age | 0.12 | 2.27 | 0.04 | 0.138 |
| SFA intake | 0.00 | 0.07 | 0.00 | 0.791 |
| **Model with ghrelin** *F*_(4,54)_=0.63, *p*=0.641, R^2^_adj._=-0.03 | | | | |
| WHR category [normal] | -0.03 | 0.11 | 0.00 | 0.745 |
| Ln ghrelin | 0.51 | 0.22 | 0.00 | 0.640 |
| Gestational age | 0.12 | 2.34 | 0.04 | 0.132 |
| SFA intake | 0.00 | 0.04 | 0.00 | 0.834 |

The model was adjusted for gestational age and saturated fatty acids. The significant associations are asterisked. SFA - saturated fatty acids; WHR- waist-to-hip ratio
